# Targeted Therapy of Antibody-Induced Autoimmune Arthritis Using Peptide-Guided Nanoparticles

**DOI:** 10.3390/ijms252212019

**Published:** 2024-11-08

**Authors:** Hemalatha Nanjaiah, Kamal D. Moudgil

**Affiliations:** 1 Research and Development, VA Maryland Healthcare System, Baltimore VA Medical Center, Baltimore, MD 21201, USA; 2 Department of Microbiology and Immunology, University of Maryland School of Medicine, Baltimore, MD 21201, USA; 3 Division of Rheumatology, Department of Medicine, University of Maryland School of Medicine, Baltimore, MD 21201, USA

**Keywords:** collagen antibody-induced arthritis, inflammation, liposomes, rheumatoid arthritis, targeted drug delivery, joint-homing peptide, nanotechnology

## Abstract

Rheumatoid arthritis (RA) is an autoimmune disease characterized by chronic inflammation of the joints and it affects over 18 million people worldwide. Despite the availability of a variety of potent drugs for RA, over 30–40 percent of patients fail to achieve adequate remission, and many patients suffer from systemic adverse effects. Thus, there is an urgent need for a joint-targeted drug delivery system. Nanotechnology-based drug delivery methods offer a promising resource that is largely untapped for RA. Using the T cell-driven rat adjuvant-induced arthritis (AA) model of human RA, we developed a peptide-targeted liposomal drug delivery system for arthritis therapy. It was based on a novel joint-homing peptide ART-2 to guide liposomes entrapping dexamethasone (Dex) to arthritic joints of rats, and this approach was more effective in suppressing arthritis than the unpackaged (free) drug. To de-risk the translation of our innovative drug delivery technology to RA patients, we undertook the validation of ART-2-liposomal delivery in a genetically and mechanistically distinct arthritis model in mice, the collagen antibody-induced arthritis (CAIA) model. Using live imaging for tissue distribution of liposomes in vivo, immunohistochemistry of paws for cellular binding of ART-2, and liposomal Dex delivery, our results fully validated the key findings of the rat model, namely, preferential homing of peptide-functionalized liposomes to arthritic joints compared to healthy joints, and higher efficacy of liposomal Dex than free Dex. These results offer a proof-of-concept for the benefits of targeted drug delivery to the joints and its potential translation to RA patients.

## 1. Introduction

Rheumatoid arthritis (RA) is a chronic autoimmune disease of the synovial joints and inadequately treated RA can result in joint damage and disability [1,2,3]. RA affects people in many countries, with the prevalence ranging from 0.25 to 1.0 percent of the population [1,2]. Women are affected two to three times more often than men. Both genetic and environmental factors play a role in RA pathogenesis. The *HLA-DRB1* gene is strongly associated with genetic predisposition to RA, and the risk to develop RA is 2- to 5-fold higher in the first-degree relatives compared to the general population. Furthermore, certain viruses (e.g., Epstein–Barr virus), cigarette smoking, obesity, and the gut and lung microbiomes are among the environmental factors linked with RA [1,2,3]. Several anti-arthritis drugs are currently available for the management of this debilitating disease. However, these drugs fail to induce disease remission in over 30–40 percent of patients, and their long-term consumption may lead to adverse effects, which can contribute to non-compliance to therapy [4,5]. One approach to overcome these limitations is to develop novel methods of drug delivery to arthritic joints. In this context, nanotechnology-based drug delivery methods offer a promising avenue that has mostly been explored for cancer therapy [6,7,8]. Liposomes are a type of nanoparticle that can entrap both hydrophobic and hydrophilic drugs inside them, and liposomal delivery can enhance the half-life as well as bioavailability of drugs with otherwise poor stability or solubility profiles [9,10]. In addition, the display of tissue-targeting ligands (e.g., antibodies or peptides) on the surface of liposomes has been shown to achieve targeted drug delivery to particular cancer tissues [11,12,13,14,15,16]. Gradually, targeted liposomal therapy approaches for various malignant tumors are being developed in preclinical models of cancer as well as tested and used in cancer patients [15,17,18]. We have adopted a similar approach for arthritis therapy using liposomes displaying a novel joint-homing peptide [19,20,21].

Using the rat adjuvant-induced arthritis (AA) model of human RA in the inbred Lewis rat (RT.1^l^), we developed a peptide-targeted liposomal drug delivery system for arthritis therapy using a novel joint-homing peptide ART-2 [19]. Liposomes displaying this peptide on their surface, when injected intravenously (i.v.) into AA rats, showed preferential homing to arthritic joints. Therefore, we used ART-2-functionalized liposomes entrapping dexamethasone (Dex) for arthritis therapy in AA rats [19]. Peptide-targeted liposomal Dex was more effective in inhibiting the progression of arthritis in rats than free Dex. However, unlike inbred rats, which are genetically homogeneous and generate immune response mainly to heat-shock protein 65 (Hsp65) during the course of AA [22], RA patients have diverse genetic make-ups and generate immune response to different target antigens, including Hsp65 and collagen type II (CII) [3,23,24,25,26]. Therefore, to further validate and de-risk the translational potential of our innovative joint-targeted drug delivery technology for RA patients, we performed independent testing of this approach in a second model of RA, the collagen antibody-induced arthritis (CAIA) model in mice [27]. The genetic and mechanistic characteristics of the mouse CAIA model are different, yet complementary, to that of the rat AA model. These two animal models of RA share some of the features of human RA, but they also differ from each other in a few critical characteristics as follows [22,27]: (a) AA is a T cell-mediated disease, whereas CAIA is a predominantly antibody-induced disease; and antibodies are pathogenic in CAIA, but either show no effect or are protective in AA; (b) unlike Lewis rats (RT-1^l^), inbred wild type mice, including DBA/1 mice (H-2^q^), do not develop AA following immunization with heat-killed *M. tuberculosis* H37Ra; (c) the predominant target of the autoimmune response is CII in CAIA, but Hsp65 in AA [22]; and (d) the metabolism of drugs might have some differences in rats and mice. A major question addressed in this study is whether the joint-homing peptide ART-2, initially characterized and tested in the rat AA model [19], can also home to arthritic joints in mice (when used as a peptide ligand for nanoparticles) and be effective in improving the therapeutic profile of an anti-arthritis drug (Dex) in a second (mouse) model of arthritis.

In this context, we undertook this study to evaluate the targeted drug delivery technology based on ART-2-liposomes in the mouse CAIA model. However, prior to testing this technology for drug delivery, we examined and validated another finding from the previous study in the rat AA model [19], namely, the binding of ART-2 peptide to CD31+ endothelial cells in arthritic joints of mice having CAIA. Our results in the mouse CAIA model described below fully validated the key findings previously observed in the rat AA model, including selective homing of ART-2-displaying liposomes to arthritic joints, the binding of ART-2 to CD31+ endothelial cells, and improved therapeutic efficacy of Dex delivered via ART-2-displaying liposomes compared to unpackaged (free) Dex. We hope that after appropriate modifications, this drug delivery platform might be suitable for use in RA patients.

## 2. Results

**Figure 1:**
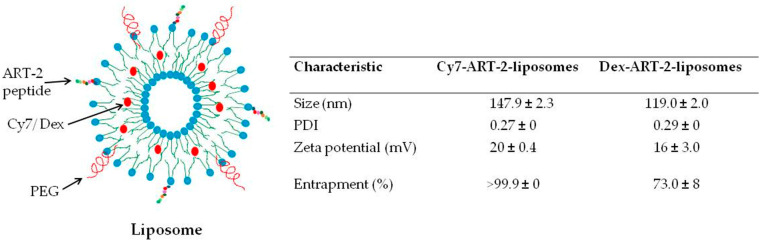
A schematic diagram of liposome composition and basic characteristics. Liposomes were prepared by thin-film hydration method. The surface of these liposomes displays a joint-homing peptide ART-2. These liposomes also have polyethylene glycol (PEG) on their surface to prevent their rapid clearance by the reticuloendothelial system. Furthermore, Cy7, a near infra-red dye, or Dex, a known anti-RA drug, is entrapped within the hydrophobic compartment of liposomes, which are referred to as Cy7-ART-2-liposomes or Dex-ART-2-liposomes, respectively. The basic characteristics of the liposomes shown include their size, polydispersity index (PDI), zeta potential, and entrapment efficiency (EE) of the cargo (dye/drug).

We prepared liposomes displaying peptide ART-2 on their surface but entrapping within them either a dye, Cy7 (Cy7-ART-2-liposomes), or a drug, Dex (Dex-ART-2-liposomes) (Figure 1). These liposomes were used for in vivo imaging and targeted therapy, respectively, of mice having CAIA.

### 2.1. In Vivo Tissue Distribution of Cy7-Entrapping Liposomes in Arthritic Animals

**Figure 2:**
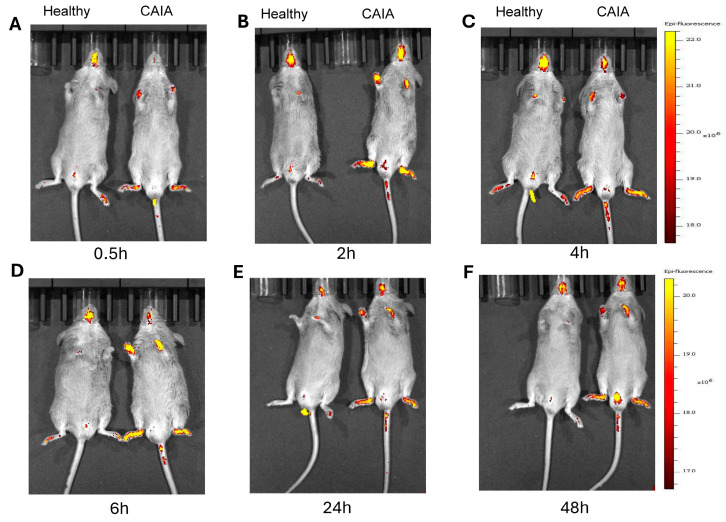
The time kinetics of in vivo biodistribution of Cy7-ART-2-liposomes in CAIA/control mice. Live images of arthritic mice vs. healthy (control) mice using IVIS (Xenogen, Alameda, CA, USA) at the indicated time points (0.5–48 h) (**A**–**F**) after i.v. injection of Cy7-ART-2-liposomes.

**Figure 3:**
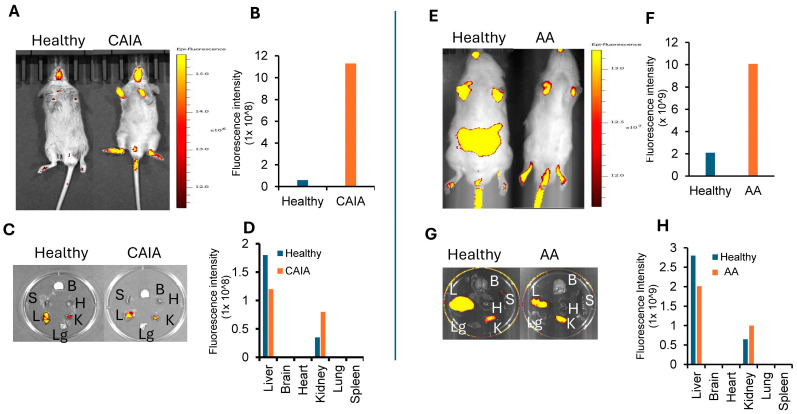
In vivo biodistribution of Cy7-ART-2-liposomes in arthritic/control animals. Live images of healthy (control) vs. arthritic animals at 4 h time point after i.v. injection of Cy7-ART-2-liposomes into CAIA mice (**A**–**D**) and AA rats (**E**–**H**) using IVIS (Xenogen) showing fluorescence signal from Cy7 ranging from dark brown to yellow, low to high. Ex vivo images of various organs harvested from these animals after 4 h of i.v. injection of these liposomes are shown: B = brain; H = heart; K = kidney; L = liver; Lg = lung; and S = spleen. color shows fluorescence signal from Cy7 ranging from dark brown to yellow, low to high.

Live imaging of animals injected i.v. with Cy7-ART-2-liposomes showed a distinct pattern of tissue distribution in arthritic animals compared to healthy (control) animals (Figure 2 and Figure 3). In CAIA mice (Figure 2), the intensity of the fluorescence signal in both hind paws and fore paws showed a gradual increase post-injection, with optimal intensity at 4 h. The fluorescence intensity at each of the time points tested was much higher in the inflamed joints of arthritic mice than that of uninflamed joints of control mice. To examine the systemic tissue distribution of Cy7-ART-2-liposomes, we examined the organs harvested from mice at 4 h time point post-injection. No fluorescence signal was detectable from the brain, heart, lung, and spleen (Figure 3A–D). However, a low-level signal was observed in the case of the liver and kidney of both CAIA and control mice, suggesting that excretion of the dye through these two organs might account for the observed signal. For comparison and reference for CAIA, the results of a similar experiment performed in arthritic (AA) rats at the 4 h time point post-injection are also shown (Figure 3E–H). We previously reported that the 4 h time point had optimal fluorescence signal strength in arthritis joints of AA rats [19], and this was confirmed again in CAIA mice (Figure 2 and Figure 3). Furthermore, the fluorescence signal in the liver and kidney of CAIA mice versus healthy mice, as well as AA rats versus healthy rats, was comparable, which in turn supports our proposition that these two organs serve as the excretion routes for Cy7. Thus, the overall tissue distribution of Cy7-ART-2-liposomes was comparable in the two models of RA tested.

### 2.2. The Binding Characteristics of ART-2 Peptide to CD31+ Endothelial Cells in Arthritic Joints of CAIA Mice

**Figure 4:**
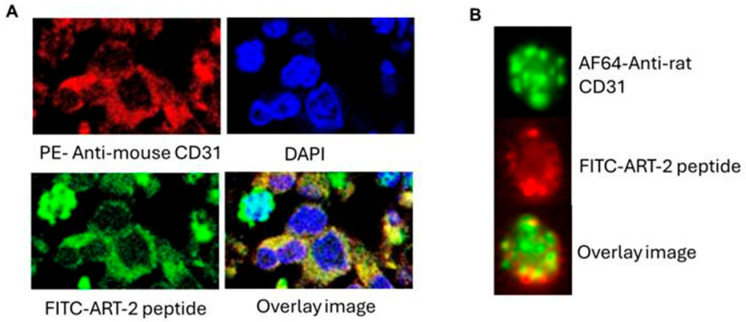
Analysis of the cellular binding of peptide ART-2 to CD31+ endothelial cells. (**A**) Immuno-histochemistry analysis of ART-2 peptide binding to endothelial cells in arthritic joints of mice. Hind paws of mice having CAIA were harvested, frozen in OCT, and processed for immunohistochemistry analysis using anti-mouse CD31 antibody conjugated with PE (red), followed by staining with peptide ART-2 conjugated with FITC (green). Also shown is staining by DAPI, a nuclear stain, and the overlay image. (**B**) Image flow cytometry analysis of ART-2 peptide binding to joint-derived endothelial cells of arthritic rats. Synovial-infiltrating cells isolated from arthritic rat joints were stained with anti-rat CD31 antibody conjugated with Alexa Fluor 647 (AF647) (red), followed by staining with peptide ART-2-FITC (green). Overlay image of the two stains is shown.

To examine the reactivity of peptide ART-2 with the CD31+ endothelial cells within the arthritic joint tissue, we performed immuno-histochemistry of hind paw sections of CAIA mice using fluorescence-labeled ART-2 peptide and anti-CD31 antibody (Figure 4A). The staining showed binding of both these reagents to a subset of cells that were CD31+ in the synovial pannus tissue. Furthermore, there was co-localization of the two stains. For reference, results of a similar staining but using flow cytometric examination of CD31+ synovial-infiltrating cells isolated from hind paws of AA rats are also shown (Figure 4B). The results of ART-2 staining in CAIA mice were comparable to those from AA rats. These results formed the basis for testing of ART-2-liposomes for drug delivery for arthritis therapy.

### 2.3. Targeted Therapy of CAIA Mice Using Dex-Entrapping Liposomes

**Figure 5:**
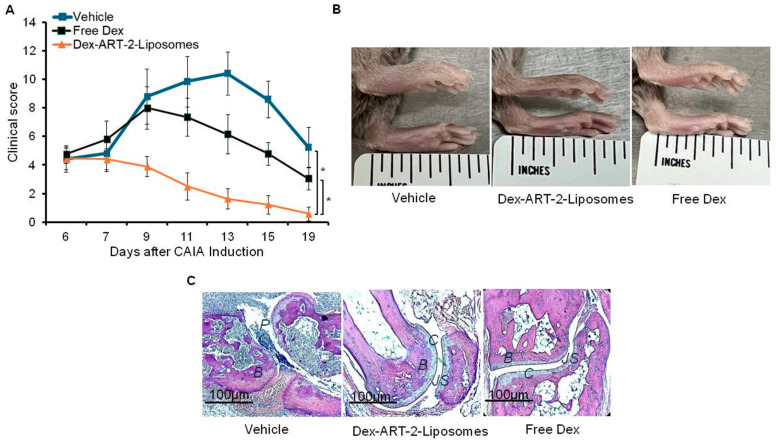
Comparative evaluation of the efficacy of Dex delivered via ART-2-liposomes versus unpackaged (free) Dex in CAIA mice. (**A**) At the onset of signs of arthritis (d 6), mice were treated i.v. either with Dex-ART-2-liposomes or free Dex, each on alternate days for a total of 5 injections. Another group of mice was treated with the vehicle and served as a control for the other two groups. The course of clinical scores of all mice is shown and the differences between clinical scores (mean ± SD) were analyzed statistically. (* = P < 0.05). Photographs (**B**) and H&E-stained sections (**C**) of hind paws of mice are also shown. In section ‘C’, the letters in italics refer to the following joint structures: B = bone; C = cartilage; JS = joint space; and P = pannus.

CAIA mice at the onset of arthritis (d 6 after disease induction) were treated i.v. either with Dex-ART-2-liposomes or with free Dex (Figure 5). Another group of CAIA mice received the vehicle on corresponding days. Our results show that arthritis severity, as measured from clinical scores (mean ± SD), was reduced by treatment with both formulations of Dex (Figure 5A,B), but the effect was more marked with liposomal Dex (P < 0.05) than that with free Dex. We also reinforced the clinical outcomes by histological examination of hind paws of different groups of mice by examining the extent of pannus formation and tissue damage within the joints (Figure 5C). As observed in our earlier study in a rat AA model [19], our preliminary results in CAIA mice also showed comparable effect of Dex delivered via plain liposomes (lacking ART-2) and free Dex. Therefore, in this study, we tested only two modalities, Dex-ART-2-liposomes and free Dex in CAIA mice (Figure 5).

## 3. Discussion

An effective control of RA symptoms and disease pathology is essential to prevent deformity and disability. Although significant advances have been made in RA therapy over the past two decades, a sizable proportion of patients have an inadequate response or are refractory to therapy. The reasons for the failure of existing therapies may differ for each treatment option, including efficacy and adverse effects. Furthermore, a proportion of treatment-refractory RA patients suffer from “difficult-to-treat” (D2T) RA [28], the designation of which has to meet specific criteria laid out by the European League Against Rheumatism (EULAR) [29]. Such patients have a higher disease burden, including a higher rate of adverse events and comorbidities, as well as a higher incidence of concomitant fibromyalgia compared to other RA patients [28,29]. The precise mechanisms underlying sub-optimal response to therapy remain to be defined. A recent study examining treatment resistance in RA patients revealed that an increase in the proportion of a subset of dendritic cells called dendritic cell precursor (pre-DC) in the peripheral blood of RA patients correlated with poor response to treatment [30]. This pre-DC subset is different from plasmacytoid DC (pDC). Furthermore, pre-DC in synovial tissue exhibits a phenotypic profile resembling that of inflammatory type-2 conventional DC (cDC2) subset. It is hoped that new approaches to modulate the expansion and activity of pre-DC might help in overcoming resistance to therapy by certain anti-arthritic drugs. In regard to the impact of treatment failure of conventional drugs, increasing numbers of patients with RA and other types of arthritis resort to natural products of diverse sources for relief of symptoms. These include natural products from plants (e.g., Tripterygium Wilfordii and resveratrol), animals (e.g., bovine collage hydrolysate), and marine organisms (e.g., sulfated glucosamine) [31,32]. The administration of above-mentioned plant products has been reported to inhibit arthritis in a mouse model [32]. Similarly, dietary supplementation with collagen hydrolysate was found to be chondroprotective in osteoarthritis in dog-patients [31]. Above-mentioned limitations highlight the challenges associated with therapeutic management of RA.

We have developed and validated a nanoparticle-based drug delivery method for arthritis therapy with the purpose of designing a platform technology that is both versatile and permits preferential targeting of arthritic joints, but not healthy joints or other organs of the body. The versatility of our technology lies in the potential of liposomes to entrap therapeutic agents of diverse types: hydrophobic or hydrophilic; small size or large size; and peptide/protein or oligonucleotides. In this study, we used liposomes entrapping Dex within them. However, our technology is not limited to liposomes. Other types of nanoparticles (e.g., micelles and polymeric nanoparticles) may also be considered for drug delivery [33,34]. We preferred liposomes because this category of nanoparticles is currently approved by the U.S. Food and Drug Administration (FDA) and other regulatory agencies globally for human use, for example, for cancer therapy [35]. In this context, liposomal formulation of the following drugs has been tested clinically in cancer patients and shown effectiveness: Doxorubicin for breast cancer, ovarian cancer, colon cancer, stomach cancer, and Kaposi’s sarcoma; Daunorubicin for AIDS-related Kaposi’s sarcoma; Cytarabine for central nervous system lymphoma and leukemia; Paclitaxel for non-small cell lung cancer; Irinotecan for pancreatic cancer; Vincristine for acute lymphoblastic leukemia; and Mifamurtide for high-grade non-metastatic osteosarcoma [15,16,17,18]. An additional application of liposomal therapy is to combine it with conventional clinical therapy, such as a combination of Caelyx and Doxil^®^ (Baxter, Deerfield, USA) for colorectal cancer [15,16,17,18].

In this study, the preferential targeting of liposomes to arthritic joints following i.v. administration is facilitated by peptide ART-2 displayed on the surface of liposomes. This targeted drug delivery to arthritic joints was aimed at directing most of the entrapped drug to the primary site of the disease, the arthritis joints, with minimal or no distribution to healthy joints or other organs of the body. The rationale for a preferential enrichment of a drug in the target organ is based on the results of live imaging of CAIA mice and ex vivo imaging of their harvested organs. The results of live imaging showed an optimal fluorescence signal emitted by Cy7-ART-2-liposomes in the hind paws of arthritic mice at different time points compared to minimal or no fluorescence signal from hind paws of healthy (control) mice. Importantly, there was barely any accumulation of the Cy7-containing liposomes in several other organs of the body, including the brain, heart, lung, and spleen. However, there was some fluorescence signal in the case of liver and kidney. Apparently, these two organs serve as the routes of excretion of the drug from the blood circulation. This is further supported by the observation that unlike the marked difference in the fluorescence signal in the paws of arthritic vs. healthy animals (CAIA mice or AA rats), the signal intensity in the liver and kidney was comparable in the two groups (mice or rats). Other investigators have also reported fluorescence signal in the liver following in vivo imaging of tumors in mice and attributed it to excretion of the dye via the liver [36].

Unlike widespread systemic distribution of a drug administered orally or by injection, the ART-2-guided drug delivery to the arthritic joints would lead to enrichment of the drug in the joints relative to other organs. Our results demonstrate that peptide ART-2-guided liposomal delivery of Dex was more effective than unpackaged (free) Dex, when used at an equivalent dose and frequency of injections in mice with CAIA. We attribute the higher efficacy of Dex-ART-2-liposomes versus free Dex to the above proposition. In a previous study in rat AA, we observed that the efficacy of Dex delivered via plain liposomes that lacked ART-2 on their surface was similar (P > 0.05) to that of free Dex [19]. Our preliminary testing in the CAIA model also showed similar results. Accordingly, in this study, detailed therapeutic testing of Dex was performed using Dex-ART-2-liposomes and free Dex only. Furthermore, the improved therapeutic profile (efficacy/toxicity ratio) of Dex in the rat AA model was primarily because of increased efficacy, but unaffected toxicity parameters (serum enzymes) [19]. Therefore, this validation study in mouse CAIA was also focused on the efficacy aspect of Dex. Taken together, the results of our study offer support for further considering this targeted drug delivery technology for eventual translation to RA patients. Moreover, extrapolating from results of our study on rat AA showing that peptide-guided liposomes containing Dex are also effective in suppressing arthritis when injected subcutaneously (s.c.), we suggest that this drug delivery approach can also be applicable to anti-arthritic drugs (e.g., methotrexate) that are currently administered to RA patients by s.c. injection [21].

The tissue-homing peptides are promising ligands that can be employed for surface-modification (or functionalization) of nanoparticles for enhancing their tissue-/organ-specificity and targeted delivery of the cargo carried within those nanoparticles. Most of the work on the identification of such peptides has been done in animal models of cancer [14,37,38], with relatively fewer studies in animal models of autoimmune arthritis [39,40,41,42,43,44,45]. For example, peptides homing to breast cancer, prostate cancer, and lung cancer have been described in mice, with each of these peptides showing specificity for a particular tissue [14,37,38]. Interestingly, in many cases, the tumor-homing peptides bound to the vascular endothelium within that tissue, indicating that the tumor vasculature expressed markers that were different from the normal vasculature of mice [14,37]. Similar studies by others [39,43] and us [19,45] in animal models of arthritis have revealed peptides homing to arthritic joints as well as targeting the endothelial cells in the synovial vasculature. In these instances, the peptides preferentially bound to the synovial vasculature of arthritic joints, but not normal healthy joint tissue. For these reasons, in this study using the CAIA model, we tested whether peptide ART-2 can bind to CD31+ endothelial cells in arthritic joints of mice. Our results validated such binding to the joint-resident CD31+ cells in the paws of CAIA mice as well as paws of AA rats. Thus, the finding in the rat model was validated in the mouse model. Furthermore, extending the interaction of peptide ART-2 with endothelial cells to human endothelial cells, we reported earlier that ART-2 binds avidly to human umbilical vein endothelial cells (HUVEC) [19]. Apparently, the putative receptor that binds ART-2 is shared among the 3 species (rat, mouse, and human) either in the form of a highly conserved protein or via a cross-reactive binding domain/site within homologous proteins. However, the identity of the precise cellular target of ART-2 remains to be determined. This work is currently in progress in our laboratory. Nevertheless, these findings are highly encouraging for translational research on peptide ART-2 as a ligand for liposomal drug delivery in human RA.

The above-mentioned joint-homing peptides have been exploited for therapy using two different approaches. First, using the peptide as a guide to direct liposomes entrapping a therapeutic agent within them, for example, Dex [19,21,40], prednisone [44], or an immunomodulatory cytokine, IL-27 [41]. Second, using constructs in which the peptide was conjugated with the therapeutic agent, for example, an apoptotic peptide [39] or an anti-inflammatory cytokine, IL-4 [46]. Similarly, other investigators have targeted folate receptors on macrophages for liposomal drug delivery for arthritis therapy [47]. However, despite the above-mentioned peptides sharing the arthritic joint-homing property [39,43,44,45], there is no consensus amino acid sequence that is common among them. Thus, it is not clear as to how these peptides home to arthritic joints. This aspect of the mechanisms remains to be determined.

The results described above offer validation of our peptide-guided drug delivery in a second model of RA. However, our study has some limitations. We have tested male DBA/1 mice in this study. Although we hoped that our results would be replicated in female DBA/1 mice, this comparison of male versus female mice remains to be undertaken. Another limitation is that at present we do not know the precise relative amount of Dex that is delivered to arthritic joints when injected in the form of ART-2-liposomes versus unpackaged (free) drug. For that, extraction of Dex from the joint tissue and its estimation by HPLC would be required. Nevertheless, the current study in the mouse CAIA model has validated our main results previously observed in the rat AA model. This ART-2-functionalized liposomal drug delivery approach for RA would add to other nanoparticle-based approaches under development for arthritis and other autoimmune diseases [36,37,38,39,40].

The desired goals of a therapeutic regimen for RA patients are to combine high efficacy with low toxicity, to enhance patient compliance with the treatment schedule, and to improve the quality of life of these patients. We believe that our novel joint-targeted drug delivery approach can help achieve these objectives. Efficacy testing of a drug in preclinical animal models is an essential requirement for advancing the testing of a new investigational new drug to RA patients. The results of this study in the mouse CAIA model have validated our previous results obtained in the rat AA model. We hope that this validation will facilitate further development of our technology for use in RA patients in the near future.

In this study, we have focused our attention on targeted drug delivery to arthritic joints. There are additional innovative ways for targeted therapy of arthritis. For example, vaccination with an antigen complex consisting of a galactosylated peptide of CII and murine class II (A^q^) has been shown to induce tolerance by targeting antigen-specific T cells and expansion of nonconventional regulatory T cells [48]. The dominant tolerance induced by this vaccination approach was effective in controlling collagen-induced arthritis (CIA) in mice. Furthermore, cartilage destruction occurs with progression of RA and OA. There is reduction in joint lubricants such as hyaluronic acid (HA) and lubricin, coupled with an increase in phospholipid species [49]. Accordingly, we anticipate that with appropriate modifications, some of the strategies for targeting arthritic joints, including joint-homing nanoparticles, might also be of utility in delivering defined lubricants into arthritic joints.

In conclusion, our results demonstrate that peptide ART-2 shows binding to CD31+ endothelial cells derived from the arthritic joints, and ART-2-liposomes injected i.v. into CAIA/healthy mice show preferential homing to arthritic joints, but not to healthy joints. Furthermore, Dex delivered i.v. at an equivalent dose to CAIA mice via ART-2-liposomes is more effective in inhibiting arthritis progression than unpackaged (free) Dex. Taken together, our results in the mouse CAIA model have validated the previously reported results in the rat AA model.

## 4. Materials and Methods

### 4.1. Preparation and Characterization of Nanoparticles

Liposomes were prepared following the thin film hydration method, using a mixture of 4 lipids, a joint-homing peptide (ART-2), and a dye or drug, as described in our previous study [19]. The lipids used were cholesterol; 1,2-dioleoyl-sn-glycero-3-phosphocholine (DOPC); 1,2-dioleoyl-sn-glycero-3-phosphoethanolamine (DOPE); and 1,2-distearoyl-sn-glycero-3-phosphoethanolamine (DSPE-(PEG) _45_-NH_2_) (Sigma Aldrich (Saint Louis, MO, USA))/Avanti polar lipids, Inc. (Birmingham, AL, USA). Peptide ART-2 was conjugated with a lipid (octadecanamine) tail (Lifetein, Somerset, NJ, USA) to enable its incorporation into the liposome membrane [19]. The dye used was cyanine 7 (Cy7) (Lumiprobe, Hunt Valley, MD, USA), which is a near infra-red dye, whereas the drug used was dexamethasone (Dex) (Sigma, Saint Louis, MO, USA), a mainstream anti-RA drug. The mixture of the above components was dried under a flow of nitrogen gas and then subjected to hydration and sonication. After separating the unencapsulated dye/drug by centrifuge-filtration, the resulting liposomes were characterized by using a Zetasizer (DLS Malvern Zeta sizer, Nano, Westborough, MA, USA) to determine their size (nm), polydispersity index (PDI), and zeta potential (mV) [41]. For quantification of entrapped Dex, the liposomes were lysed by methanol and the released Dex was measured by high performance liquid chromatography (HPLC) using a C18 column (Waters Inc., photodiode detector Milford, MA, USA). The amount of entrapped Dex was estimated and presented as entrapment efficiency (%) (EE = (Wt − Wf)/Wt × 100; Wf is the amount of free drug not incorporated in the liposome suspension and Wt is the total quantity of drug added to the lipid mixture). The liposomes containing Cy7 or Dex are referred to as Cy7-ART-2-liposomes or Dex-ART-2-liposomes, respectively, whereas similar liposomes but lacking peptide ART-2 on their surface constitute ‘plain liposomes’.

### 4.2. Collagen Antibody-Induced Arthritis (CAIA) Model in Mice

Male DBA/1 mice (H-2^q^), 7–8 wk old, were injected intraperitoneally (i.p.) with a mixture of anti-collagen type II (CII) antibodies (Chondrex, Woodinville, WA, USA) on day 0, followed by i.p. injection of lipopolysaccharide (LPS) on day 4, as per the manufacturer’s instructions. Thereafter, mice were observed regularly for redness or swelling of hind paws and fore paws. A grading scale of 0 to 4 per paw was used to determine the severity of arthritis as follows [19,50]: 0 = no erythema or swelling of ankle/wrist; 1 = erythema and swelling limited to the ankle/wrist; 2 = erythema and swelling of the ankle/wrist and the tarsus/carpus, respectively; 3 = erythema and swelling involving the ankle/wrist, the tarsus/carpus, and the metatarsus/metacarpus, respectively; and 4 = swelling of the whole hind paw/fore paw, including the digits. In some cases, fractional scores (e.g., 0.5, 1.5) were added to the above scheme to account for signs that were not fully developed to use a particular whole number grade. Besides clinical scoring, histopathological examination of the hind paw sections stained with hematoxylin and eosin (H&E) was performed to confirm changes relating to arthritic inflammation and tissue damage in the joints [19]. Prior approval of the Institutional Animal Care and Use Committee (IACUC) of University of Maryland, Baltimore was obtained before performing any animal experimentation. This research work was also approved by the Review Boards of Veterans Affairs.

### 4.3. Live Imaging of CAIA/Healthy Mice and Ex Vivo Imaging of Their Harvested Organs

Cy7-ART-2-liposomes were used for in vivo imaging of arthritic mice. These liposomes were injected i.v. into either arthritic mice after the onset of CAIA or healthy (control) mice. Live imaging of these mice was performed under anesthesia using IVIS (Xenogen) equipment (Perkin Elmer, Hopkinton, MA, USA) at different time points post-injection [19]. Thereafter, following euthanasia, various organs of these mice were harvested and ex vivo imaging was performed on them. The fluorescence signal intensity of images was quantified from the specified region of interest (ROI).

### 4.4. Treatment of CAIA Mice with Liposomal Dex

CAIA was induced in a cohort of DBA/1 mice as described above. At the time of onset of signs of arthritis (6 days after disease induction), mice were randomized and separated into groups for treatment. The two experimental groups (*n* = 10 each) were treated i.v. either with Dex-ART-2-liposomes or with unpackaged (free) Dex. An equivalent dose of Dex (1.5 mg/kg) was used for these two formulations. This dose was selected after performing pilot tests. The control group (*n* = 10) was treated i.v. with the vehicle. Injections were given on alternate days and a total of 5 injections were given. All mice were euthanized on d 19 after disease induction and their hind paws were harvested and processed for histopathology or immunohistochemistry.

### 4.5. Immunohistochemistry (IHC) of Hind Paws of CAIA Mice

The frozen cryotome sections prepared from optimal cutting temperature (OCT) compound-embedded tissue were fixed with 4% paraformaldehyde. This was followed by washing and blocking of the non-specific binding sites and staining with fluorescence isothiocyanate (FITC)-labeled ART-2 peptide and phycoerythrin (PE)-labeled anti-CD31 antibody for 2 h at room temperature. At the final step, the tissue was layered with a mounting medium (Med Chem Express, Monmouth Junction, NJ, USA) containing 4′,6-Diamidino-2-phenylindole dihydrochloride (DAPI), which is a nuclear stain, and then covered with a cover slip and viewed under a microscope (Carl Zeiss Confocal Microscope LSM700, ZEISS, White Plains, NY, USA).

### 4.6. Induction of AA in Rats and Live/Ex Vivo Imaging of Rats/Organs, Respectively

AA was induced in Lewis rats (Envigo, Indianapolis, IN, USA) by subcutaneous (s.c.) immunization with heat-killed *M. tuberculosis* H37Ra (Mtb) (Difco Laboratories, Detroit, MI, USA) in mineral oil. These rats were then observed for arthritis development using a grading scale of 0–4 per paw as described in detail elsewhere [19]. After the onset of AA (about 9–11 d after Mtb injection), following the procedure described above for CAIA mice, live imaging (IVIS, Xenogen, Perkin Elmer, Hopkinton, MA, USA) of AA rats and healthy (control) rats was performed after i.v. injection of Cy7-ART-2-liposomes. In addition, upon termination of the experiment, the harvested organs of rats were examined ex vivo using the same imaging equipment.

### 4.7. Assessing the Binding of Peptide ART-2 to CD31+ Endothelial Cells Isolated from Arthritic Rat Joints Using Flow Cytometry

The synovial-infiltrating cells were harvested from the ankle joints of arthritic rats and passed through a cell-strainer (70 µm pore size) (MTC Bio, Sayreville, NJ, USA). These cells were treated with a blocking buffer, followed by sequential staining with anti-rat CD31 conjugated with Alexa Fluor 647 (Bio-Rad, Hercules, CA, USA) and peptide ART-2 labeled with FITC (Gen Script (Piscataway, NJ, USA)/Lifetein, Somerset, NJ, USA) for 45 min each at room temperature. After washing, the cells were fixed with paraformaldehyde solution and the images of stained cells were captured using an imaging flow cytometer (Amnis image stream X MK II imaging flow cytometer, Amnis, Seattle, WA, USA).

### 4.8. Statistical Analysis

The data were analyzed using Wilcoxon rank-sum test or Student’s *t*-test, as applicable, and P < 0.05 was set as the cut-off to consider the difference between any two groups to be statistically significant.

## Data Availability

Data are contained within the article.
